# To the Subscribers of the Buffalo Medical Journal

**Published:** 1858-05

**Authors:** Sanford B. Hunt


### EDITORIAL DEPARTMENT.

---

*To the Subscribers of the Buffalo Medical Journal.*—Three years since I assumed the ownership of this Journal, and also the sole editorial conduct. The experiment was pecuniarily successful for the two first years, and my relations with subscribers such as were pleasant. Events have since occurred which have withdrawn me entirely from the practice of medicine, the labor involved in other duties has compelled me to tender a reluctant resignation of my Professorship in the Medical Department of the University of Buffalo, and for some months past my only connection with the profession of medicine has been through this Journal. It is not fitting that I should assume to be a teacher, or a mouthpiece of opinion, in a profession wherein I do not work.

In view of this, my connection with the Buffalo Medical Journal, both as editor and proprietor, ceases with the present number. Arrangements have been made for its continuance under the competent editorial management of Dr. Austin Flint, Jr., who has, in fact, had its exclusive control for several months past. During that period he has evinced a capacity for his position which does him honor and renders the Journal, in my opinion, the most desirable monthly in the country for the general practitioner.

The accounts of the old concern remain in my hands. Bills are enclosed in this number to all who are indebted to me, and those who find no bill may take it for granted that they owe me nothing. It is my right to insist on the prompt remittance of the amounts called for by these bills. During the past year, disastrous to all pecuniary enterprises, the Journal has been published by me with promptness and regularity at a heavy personal loss. I expected this when it became evident that a financial crisis was impending, but my obligations to advance paying subscribers compelled me to go on in the face of difficulty. I have paid my printers month by month and owe them nothing, but have been seriously embarrassed by doing so. I

now ask those indebted to me to make good my losses by prompt payment of the small amounts due from each. Last year it was impossible for them to do this—now no such excuse exists. It is better for the Journal that I should retain these accounts, as it saves the new proprietor the annoyance of the old bills, and enables subscribers to start anew with no embarrassments from old arrearages. Remittances on old accounts must therefore be made to myself only.

And now, business set aside, I cannot part with my old readers without a word. It is five years since I became connected with the Journal. I came to it fresh from rural practice, ambitious and hopeful. All those hopes and ambitions have been realized—to an extent far more than I deserved. Yet I have turned aside to other paths, at first slightly wandering, then gradually widening the distances. For a time I tried to do too many things, and went about with the text "Unstable as water, thou shalt not excel!" ringing hourly in my ears. Now I am anchored at the editorial desk of a daily paper, and there I hope to remain, with an influence no longer weakened by division.

During this five years, however, I have labored with industry and such consistency as was natural to me, to elevate the legitimate profession of medicine, to honor its noble achievements and vindicate its high claim upon the respect and confidence of community. Sustained by a band of contributors of such talent, learning, and honorable reputation as few other medical periodicals can boast, it has been no difficult task to present a good journal. Here at home, Flint, Hamilton, White, Rochester, Newman, Nichols and others, particularly among the membership of the Buffalo Medical Association, have lent such aid as I should be very grateful for. Under the new management all these will continue to contribute, and with them many others abroad who will be equally welcome. Thus a claim is established upon the confidence of the profession which should be met with a prompt and cordial support.

To my old subscribers, who have wintered and summered with the Journal for years, I can only say in parting that I am now as ever,

Cordially their Friend,

SANFORD B. HUNT.

